# Innovative Method for Longer Effective Corrosion Inhibition Time: Controlled Release Oil Palm Empty Fruit Bunch Hemicellulose Inhibitor Tablet

**DOI:** 10.3390/ma14195657

**Published:** 2021-09-28

**Authors:** Nur Izzah Nabilah Haris, Shafreeza Sobri, Yus Aniza Yusof, Nur Kartinee Kassim

**Affiliations:** 1 Institute of Advanced Technology, Universiti Putra Malaysia, Selangor 43400, Malaysia; nurizzahnabilah.haris@gmail.com; 2 Department of Chemical and Environmental Engineering, Faculty of Engineering, Universiti Putra Malaysia, Selangor 43400, Malaysia; 3 Department of Process and Food Engineering, Faculty of Engineering, Universiti Putra Malaysia, Selangor 43400, Malaysia; yus.aniza@upm.edu.my; 4 Department of Chemistry, Faculty of Science, Universiti Putra Malaysia, Selangor 43400, Malaysia; kartinee@upm.edu.my

**Keywords:** controlled release tablet, corrosion inhibitors, weight loss, electrochemical test

## Abstract

This study aims to develop a controlled release oil palm empty fruit bunch hemicellulose (EFB-H) inhibitor tablet for mild steel in 1 M HCl. As plant extracts tend to deteriorate at longer immersion time, limiting its industrial applicability, we attempted to lengthen the inhibition time by forming a controlled release inhibitor tablet. Electrochemical methods (potentiodynamic polarization (PDP) and electrochemical impedance spectroscopy (EIS)) were employed to investigate the efficiency and mechanism of the inhibition. An optimum dosage and immersion time was determined via Response Surface Methodology (RSM). EFB-H tablet was formulated using D-optimal mixture design, and its anticorrosion action at extended immersion time was compared with EFB-H powder. PDP measurement revealed that EFB-H is a mixed type inhibitor. RSM optimization unveiled that the optimum point for a maximum inhibition efficiency (87.11%) was at 0.33 g of EFB-H and 120 h of immersion time. Tablet T3 with EFB-H to gum Arabic to hydroxypropyl methylcellulose ratio of 66:0:34 portrayed the best tensile strength (0.243 MPa), disintegration time (152 min) and dissolution behavior. EFB-H tablet exhibited a longer-lasting inhibition effect than powder, which was 360 h as compared to 120 h for powder. Overall, EFB-H tablet has been successfully developed, and its enhanced effective inhibition time has been experimentally proven.

## 1. Introduction

Corrosion inhibitors are substances that can minimize or eliminate metal corrosion once added to a corrosive solution. It is probably the most straightforward, inexpensive and efficacious strategy employed on a reasonably regular basis in the industrial sector [1]. Traditional inorganic inhibitors such as chromates, nitrates, tungstates and molybdates offer the best corrosion inhibition capability but they possess several shortcomings. A great majority of inorganic inhibitors possess toxicological issue, and thus the use of some inorganic inhibitors is now restricted, except in certain situations when human safety is on the line, such as in the aeronautical industry [2]. In addition, inorganic inhibitors are highly water soluble and do not work well in highly acidic solutions as they cannot be adsorbed on the metal surface. Furthermore, the presence of inorganic inhibitors in corrosive solutions will generate toxic gases as the metal starts to corrode [3]. Accordingly, despite the inorganic inhibitors being unarguably potent in inhibiting corrosion, environmentally friendly organic inhibitors should be explored.

Plants-based inhibitors, particularly those that are biomass-based, have emerged as potential alternatives to conventional inhibitors. To date, numerous works have attempted to utilize plant products to mitigate the corrosion of steel in various media. Gouamid et al. [4] reported that date palm leaves hemicellulose at 500 ppm achieved 89.25% inhibition efficiency for mild steel in H_2_SO_4_ solution. A study by Guedes et al. [5] has demonstrated that coconut husk extract at 1.2 gL^−1^ could inhibit approximately 90% of carbon steel corrosion in a neutral water solution. Komalasari et al. [6] investigated the use of tea and coffee leaves extracts as inhibitors for carbon steel corrosion in HCl solution. The findings revealed that 1.5 gL^−1^ of tea leaves extract and 1.5 gL^−1^ of coffee leaves extract resulted in the highest inhibition efficiencies of 79% and 52%, respectively. A recent study by Lima and co-authors [7] obtained 85.2% of inhibition efficiency with 0.8 gL^−1^ of aqueous soybean extract for mild steel in H_2_SO_4_ solution. El-Hashemy et al. [8] reported that 500 ppm of *Calendula officinalis* flower heads extract can inhibit up to 94.88% of mild steel corrosion in HCl medium. According to these reports, the capability of natural inhibitor to mitigate corrosion is undeniable.

Upon having a proper inhibitor source, there is still uncertainty whether the inhibitor is suitable for industrial application. This is due to the biodegradability of natural inhibitors, which causes their performances to deteriorate at longer immersion time, especially after 120 h of usage [9,10,11]. Even though degradation is a positive feature from an environmental point of view, it is a huge drawback for implementing natural inhibitors in the industry. Several studies have tested incorporating plant-based inhibitors in polymer coatings, which can extend the shelf life of the inhibitor [12,13]. However, this solution only applies to metal parts that can be coated. The industrial applicability of plant-based inhibitors for non-coated metal hitherto remains unresolved. A proposed solution to address this matter is to develop a controlled release inhibitor tablet. This idea was triggered based on the concept of pharmaceutical controlled release tablets and a patented invention of controlled release tablet for wastewater treatment. A controlled release inhibitor tablet is capable of releasing active compounds in a sustained manner, preventing the active compounds from degrading when in contact with corrosive environment, and hence extending the shelf life upon usage. Surprisingly, the use of controlled release tablets to combat plant-based inhibitors’ degradation issue has not been closely examined. Based on the literature, only one patented and commercialized invention focused on applying controlled release tablets in the corrosion field. The invention provided tablet formulations composed of biofilm dispersant, scale inhibitor, biocide, inorganic corrosion inhibitor and polymers for treating industrial water systems [14]. The resulting 70 mm diameter tablet was reported to have a controlled release span of up to 6 months, which successfully omitted the requirement of daily dosing in the water treatment system, eliminated liquid chemical pump usage, reduced labor, operation and maintenance cost, and provided a safer working environment.

Oil palm empty fruit bunch (OPEFB) is a fibrous material from the empty stalks of fresh fruit bunches. Conventional oil palm milling involves five primary operations: fruit sterilization, fruit loosening/stripping, fruit digestion, oil extraction and oil clarification [15]. The oil palm fruits are removed from the fresh fruit bunches during the fruit stripping process, leaving EFB as a waste product. EFB is categorized as lignocellulosic biomass as it composed of cellulose, hemicellulose and lignin. As with other plant materials, OPEFB constitutes various components that can be categorized into several groups: structural components, extractive compounds, inorganic compounds (ash) and moisture. The structural make up of OPEFB cell walls, lignocellulose, consists of a complex matrix of cellulose, hemicellulose and lignin. In our previous study, we have discovered the outstanding performances of OPEFB raw fiber and extract as corrosion inhibitor for mild steel in acidic solution, which was primarily due to the existence of various chemical moieties in its structure [16,17].

The present work aims to develop a controlled release OPEFB hemicellulose (EFB-H) inhibitor tablet for mild steel in 1 M HCl. The specific objectives targeted to achieve this goal are as follows:

1. To characterize the functional groups and chemical constituents of EFB-H.
2. To investigate the mechanism and kinetics of EFB-H anticorrosion action using potentiodynamic polarization (PDP) and electrochemical impedance spectroscopy (EIS).
3. To optimize the inhibition efficiency of EFB-H using response surface methodology-central composite design (RSM/CCD) at different dosages and immersion times.
4. To formulate EFB-H tablet using D-optimal mixture design, determine the tablet properties and compare its anticorrosion action with EFB-H powder inhibitor at extended immersion times.

## 2. Materials and Methods

### 2.1. Materials

The OPEFB used in this study was collected from Labu Palm Oil Mill located in Selangor, Malaysia. Chemical reagents such as HCl, acetone and ethanol were supplied by R&M Chemicals, Ever Gainful Enterprise Sdn. Bhd. (Selangor, Malaysia). Mild steel specimen with a chemical composition given in Table 1 were purchased from WGS Steel Sdn. Bhd. (Selangor, Malaysia).

### 2.2. Preparation of EFB-H Inhibitor

**Figure 1:**
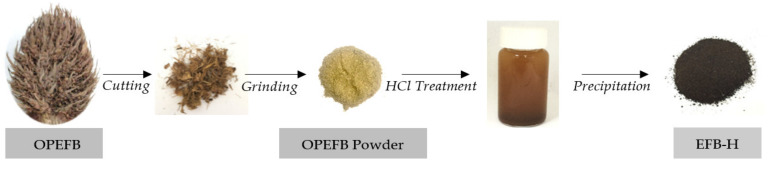
General flow of EFB-H preparation.

The EFB-H was prepared in accordance to a typical mild acid hydrolysis method with minor adjustments [18,19,20]. The preparation began with soaking raw OPEFB in 300 mL of 1 M HCl at a mass to volume ratio of 1:30. The mixture was stirred continuously at 100 rpm and heated at a temperature of 90 °C for 60 min. The resulting mixture of solid and liquid was then cooled down to 40 °C and filtered by vacuum filtration through a filter paper (11 μm). The filtrate was precipitated by adding 500 mL of 95% ethanol, left for 24 h for complete precipitation and filtered again to recover the precipitate. The precipitate was then washed with ethanol and dried at 90 °C. Figure 1 depicts the general flow of EFB-H preparation.

### 2.3. Characterization of EFB-H

#### 2.3.1. Fourier-transform Infrared Spectroscopy Analysis

The FTIR analysis was performed using an FTIR spectrometer (Perkin Elmer, Spectrum 100, Waltham, MA, USA) equipped with an attenuated total reflectance (ATR) sampling accessory (Pike Technologies, Fitchburg, WI, USA). The crystal material used in the analysis was germanium (Ge). The EFB-H sample was scanned over the range of 4000 cm^−1^ to 650 cm^−1^ with a resolution of 4 cm^−1^.

#### 2.3.2. Chemical Composition (Cellulose, Hemicellulose, Lignin and Extractives)

The chemical composition of EFB-H waste hydrolysate in terms of its cellulose, hemicellulose, lignin and extractives content was analyzed according to Technical Association of Pulp and Paper Industry (TAPPI) T222, TAPPI T249 and NREL TP-510-42619 standard procedures [21].

### 2.4. Electrochemical Tests

#### 2.4.1. Potentiodynamic Polarization

**Figure 2:**
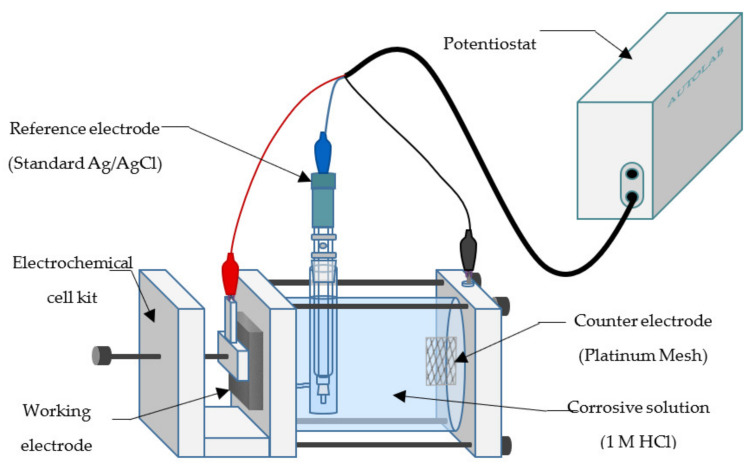
Schematic diagram of electrochemical test.

All electrochemical tests were performed using an Autolab potentiostat (Metrohm, PGSTAT101, Herisau, Switzerland) equipped with Nova Software (Metrohm, Version 1.0, Herisau, Switzerland). The experimental set up of an electrochemical cell kit with a three-electrode assembly is depicted in Figure 2. The working electrode is the mounted mild steel coupon with 0.79 cm^2^ of exposed surface area to the electrolyte. A platinum mesh was used as the counter electrode, while a standard Ag/AgCl electrode was used as the reference electrode. The cell was filled with 300 mL of corrosive solution, 1 M HCl solution with and without the addition of EFB-H at varying dosages. After the electrochemical cell has been set up, the system was allowed to stabilize and reach a steady open circuit potential for 30 min [22,23]. This step is crucial to ensure an accurate measurement as the corrosion reaction fluctuates at the initial stage [24]. After the system has stabilized, the potentiodynamic polarization measurement was performed with polarization sweep within ±0.1 V of the open circuit potential at a scan rate of 1 mVs^−1^ [25]. Each measurement was repeated three times, and only the average values were reported. New sets of corrosive solutions and mild steels were used for each repetition as the polarization measurement is a destructive technique that alters the electrode surface [26]. After completing the measurement, the data was plotted into a Tafel curve, and the electrochemical parameters such as the corrosion potential, Tafel slopes, corrosion current, corrosion rate and inhibition efficiency were computed. The corrosion potential can be computed from the lowest point of the current density in the Tafel plot, whereas the slope can be obtained by merely measuring the slope of each linear segment of the curve. As the corrosion current is known, the corrosion rate and the inhibition efficiency can be calculated using the following equations:

(1) $$C_{R}=k\frac{i_{corr}}{d}\;EW$$

(2) $${IE}_{PDP}=\frac{i_{corr}\;{-\;i}_{corr}^{inh}}{i_{corr}}\;\times \;100$$

where *C_R_* is the corrosion rate, *k* is a unit conversion factor (0.13), *d* is the density of mild steel, *EW* is the equivalent weight of iron, *IE_PDP_* is the inhibition efficiency derived from PDP method and $i_{corr}^{inh}$ is the corrosion current density of inhibited system.

#### 2.4.2. Electrochemical Impedance Spectroscopy

The EIS measurement was conducted using the same equipment, software and experimental set up as the PDP method. After reaching a steady-state, the EIS was performed within 100 kHz to 100 mHz of frequency and an amplitude of 10 mV [27]. The measurements were repeated three times, and the average data was taken. The measured data was plotted into a Nyquist plot and fitted into an equivalent circuit. Ultimately, the parameters such as the charge transfer resistance, solution resistance, double layer capacitance and inhibition efficiency were calculated. The charge transfer resistance value was obtained from the Nyquist plot semicircle curve’s diameter, while the solution resistance was measured graphically from the origin of the x-axis to the initial point of the semicircle. The following equation is used to compute the double layer capacitance:

(3) $$C_{dl}=\frac{1}{\omega_{max}\;R_{ct}}$$

where *C_dl_* is the double layer capacitance, *ω_max_* is the frequency at which the real impedance is at a maximum (top of semicircle) and *R_ct_* is charge transfer resistance. Finally, the inhibition efficiency can be calculated using the following formula:

(4) $${IE}_{EIS}=\frac{R_{ct}^{inh}{-\;R}_{ct}}{R_{ct\;}^{inh}}\;\times \;100$$

where *IE_EIS_* denotes the inhibition efficiency derived from EIS method and${\;R}_{ct}$ and $R_{ct\;}^{inh}$ are the charge transfer resistance for uninhibited and inhibited system, respectively.

### 2.5. Optimization—Response Surface Methodology

The RSM/CCD optimization was performed by Design Expert software (Stat-Ease Inc., Version 7.1, Minneapolis, MN, USA). Prior to designing an experiment, a preliminary study was conducted using one factor at a time (OFAT) method to identify suitable ranges of EFB-H dosage and immersion time. Two sets of OFAT-based experiments were performed:

1. Varying dosages of 0.1, 0.3 and 0.5 g at a fixed immersion time at 120 h,
2. Varying immersion times of 24, 120 and 216 h at a constant dosage.

All runs were conducted according to standard weight loss measurement method. Mild steel coupons were weighed to obtain the initial weight. Subsequently, each coupon was immersed in 300 mL of uninhibited and inhibited 1 M HCl solutions for 120 h at 25 °C. After immersion, each mild steel coupon was retrieved from the corrosive solution and rinsed using distilled water. Afterwards, the sample was air-dried to remove moisture and reweighed to acquire the final weight. Experiments were performed in triplicate, and average values were taken. The preliminary result was used to determine an appropriate range of variables with the highest possibility of finding an optimum response level. This step is important to avoid unnecessary experimental runs. The decided range was then specified in the software, and a set of experimental runs was generated [28]. Eventually, the software generated a mathematical expression representing the whole system within the variable ranges. The model was then used to predict an optimum level of EFB-H dosage and immersion time that yield the highest inhibition efficiency. Ultimately, the optimum point was validated experimentally to ensure the model’s prediction’s accuracy and validity.

### 2.6. Preparation and Corrosion Inhibition Study of EFB-H Tablet

#### 2.6.1. EFB-H Tablet Formulation—Mixture Design

The EFB-H tablet was formulated with the aid of D-optimal design, Design Expert software (Stat-Ease Inc., Version 7.1, Minneapolis, MN, USA). Two polymers supplied by R&M Chemicals, Ever Gainful Enterprise Sdn. Bhd. (Selangor, Malaysia), namely gum Arabic (GA) and hydroxypropyl methylcellulose (HPMC), were included in the formulation as binders to induce a controlled release behavior of the resulting tablet. The EFB-H powder weight was remained constant at an optimum level according to RSM optimization (0.33 g), which is 66% on the basis of 0.5 g tablet total weight. In contrast, the ranges of GA and HPMC were varied from 0% to 34%. These conditions were specified in the software, and a set of experimental design was generated. The tablets of varying formulations were prepared as described in the section that follows, and weight loss measurement was conducted at 120 h of immersion time with three replications. The formulation of selected tablets for further properties analysis are summarized in Table 2.

#### 2.6.2. EFB-H Tablet Compression

**Figure 3:**
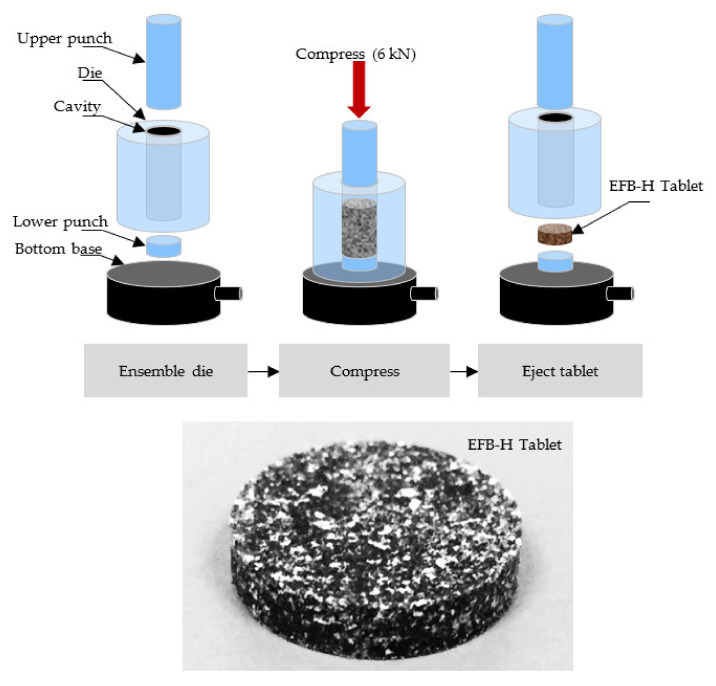
Schematic diagram of uniaxial die compression and compressed EFB-H tablet.

The EFB-H tablet compression was conducted using a universal testing machine (Instron, 5566, Norwood, MA, USA) with a maximum allowable force of 10 kN [29]. Firstly, mixtures of EFB-H, GA and HPMC powders were prepared, each at 0.5 g of total weight according to the designed formulation. The powders were thoroughly mixed and subsequently poured into a 13 mm uniaxial die (Specac, PT. No. 3000, Orpington, UK). Then, the die was compacted with 6 kN of compression force at a compression speed of 1 mm s^−1^. Eventually, the tablet was ejected from the die and stored in a desiccator until further use. The compression process and the actual compressed tablet are illustrated in Figure 3.

#### 2.6.3. Tablet Properties

The tensile strength test of the EFB-H tablet was performed using a diametrical compression test using the same instrument used for tableting [29]. The test was conducted after 24 h of tablet compression to allow the tablet to achieve a stable-state. A tablet was placed between two flat plates of the instrument and compressed diametrically until it broke. The force upon breakage was recorded, and the tensile strength was calculated. The following equation is used to determine the tensile strength of a tablet;

(5) $$T=\frac{2\;F}{\pi \;D\;T}$$

where *T* is the tensile strength, *F* is the load at fracture, *D* is the tablet diameter and *T* is the tablet thickness.

The disintegration time of the tablet was determined using a jar test apparatus according to an established pharmaceutical method with some modifications to suit corrosion inhibition application [30]. 1 M HCl was used as the dissolution medium to represent actual corrosion application. An EFB-H tablet was placed in 500 mL of the dissolution medium and agitated at 100 rpm at 25 °C. The time required for the tablet to dissolve completely was recorded.

The dissolution profile of EFB-H tablet was analyzed using a jar test apparatus, referring to a typical method for pharmaceutical tablets with some alterations to suit corrosion inhibition application [30]. Initially, a pre-weighed EFB-H tablet was placed in a mesh and positioned inside a beaker containing 500 mL of disintegration medium (1 M HCl). The solution was agitated at 100 rpm and 25 °C. Every 10 min interval, the tablet was taken out from the beaker and dried by blotting tissue paper on the tablet’s surface. The weight of the tablet was recorded and placed back into the vessel immediately. The dissolution profile was determined based on the erosion rate of the tablet. The erosion of the tablet was determined using the following expression;

(6) $$E=\frac{w_{0\;}{-\;w}_{1}}{w_{0}}\;\times \;100$$

where *E* is the erosion, *w*_0_ is the weight of tablet before immersion and *w*_1_ is the weight of tablet after immersion at each interval.

#### 2.6.4. Extended Weight Loss Study

The extended weight loss study for EFB-H tablet inhibitor was carried out according to the standard weight loss method described earlier at immersion times of 120, 240, 360, 480, 600 and 720 h.

## 3. Results

### 3.1. EFB-H Characterization

#### 3.1.1. Fourier-Transform Infrared Spectroscopy Analysis

**Figure 4:**
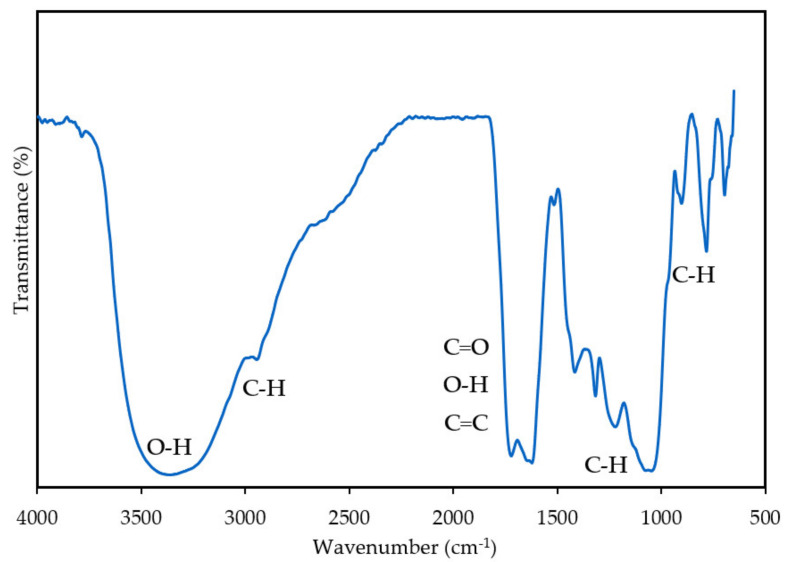
FTIR spectrum of EFB-H.

Figure 4 illustrates the FTIR spectrum of EFB-H. A strong and broad peak at 3352 cm^−1^ indicates O–H stretching vibration in alcoholic and phenolic hydroxyl groups in cellulose, hemicellulose and lignin [31,32]. The absorption peak at 2930 cm^−1^ is due to C–H stretching of methyl and methylene groups in all components of lignocellulose, but most significant in cellulose [33,34]. The bands that appeared at 1720 cm^−1^ represents the vibration of hemicellulose’s C=O uronic and acetyl groups and/or hemicellulose’s and lignin’s ester linkage of the carboxyl and p-caumaric acid [35]. The band observed at 1650 cm^−1^ corresponds to O–H stretching due to adsorbed water [36,37,38]. The presence of 1620 cm^−1^ and 1510 cm^−1^ peaks is attributed to C=C stretching of benzene ring in lignin structure [39,40,41]. The peaks located within 1315 to 1331 cm^−1^ are attributed to C–H stretching from cellulose and hemicellulose [42]. The bands that appeared at 897 to 901 cm^−1^ are assigned as C–H or CH_2_ bending vibrations, indicating the presence of β-glycosidic linkages; a bond that joins two different types of monosaccharides. This bond is a typical structure of cellulose and hemicellulose [43,44,45]. It is well known that these heteroatoms and functional groups contribute to excellent inhibitory properties [46,47].

#### 3.1.2. Chemical Composition (Cellulose, Hemicellulose, Lignin and Extractives)

The structural compositions of raw OPEFB and EFB-H on weight bases are summarized in Table 3. Based on the data, it is obvious that the holocellulose in EFB-H was higher than the raw OPEFB. EFB-H predominantly composed of hemicellulose (89.51%) and a minimal amount of cellulose (0.85%). This finding revealed that the acid treatment had disrupted the holocellulose structure, particularly hemicellulose. The relatively high dissolution of hemicellulose compared to cellulose is attributable to its structure, amorphous properties, high polydiversity and low degree of polymerization [48]. In terms of lignin recovery, approximately more than half of the acid soluble and acid insoluble lignin was recovered from the raw OPEFB. Acid soluble lignin was present in EFB-H as the lignin fraction is soluble in acid solution. This type of lignin is also normally found in acid treated lignocellulosic materials [49]. Acid insoluble lignin’s existence in a comparatively lower proportion is not unprecedented, as acid treatment also causes the breakdown and reorganization of the lignin structure [48]. In terms of the extractives, almost half of the amount extractives in the untreated sample were found in EFB-H. Extractives of plants are composed of various phytochemicals soluble in a wide range of polar and non-polar solvents [50]. Hence, compounds that are soluble in acid solution will technically present in EFB-H. Overall, it can be deduced that EFB-H is primarily composed of hemicellulose.

### 3.2. Electrochemical Tests

#### 3.2.1. Potentiodynamic Polarization

**Figure 5:**
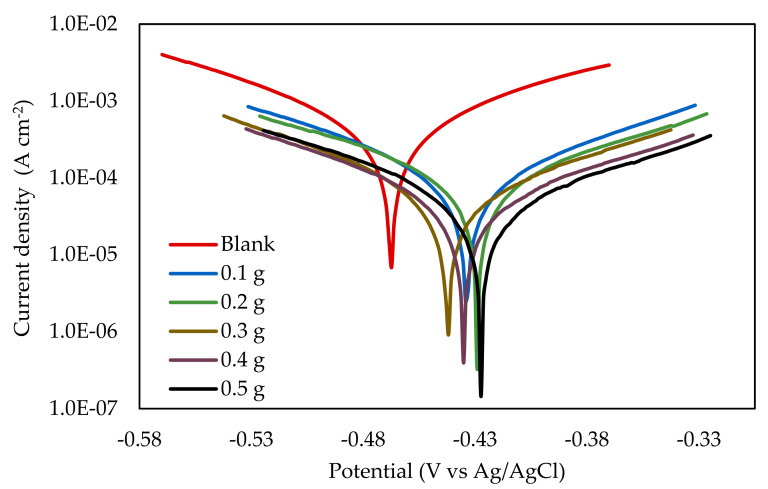
Potentiodynamic polarization curves of mild steel in 1 M HCl at different EFB-H dosages.

Potentiodynamic polarization (PDP) measurement was performed to unravel the kinetics of the electrochemical reaction of mild steel corrosion in the absence and presence of EFB-H. The Tafel polarization plot of mild steel at different dosages of inhibitor is depicted in Figure 5, whereas the kinetic parameters extracted from extrapolation of the plot such as the corrosion potential, Tafel slopes, corrosion current density, corrosion rate and inhibition efficiency are summarized in Table 4.

The corrosion potential is a valuable parameter to predict the corrosion damage in a system as well as to evaluate the effectiveness of a protective film. The corrosion potential of the mild steel specimen immersed in blank 1 M HCl solution relative to Ag/AgCl reference electrode was found to be −463.51 mV, which is within the range of −450 mV to −465 mV reported in previous studies with similar experimental conditions [51,52,53]. In the presence of EFB-H, the corrosion potentials had slightly increased by 42 mV on average. Even though the change is minimal, it indicates that the inhibited system preferentially takes up electrons through reduction reaction than losing electrons via anodic reaction. In consequence, the dissolution of metal, which is governed by the anodic reaction is less likely to occur.

The Tafel line is helpful to determine the type of inhibitor by observing the anodic and cathodic branches change with the incorporation of an inhibitor. Based on the data, the anodic and cathodic branches have shifted to lower current densities upon the addition of EFB-H. This trend suggests that EFB-H inhibits corrosion by retarding both reactions simultaneously [54]. Despite the slight increase of corrosion potentials by 42 mV, the inhibitor is considered as a mixed type inhibitor as the potential increase was lower than 85 mV [55]. Accordingly, EFB-H is categorized as a mixed-type inhibitor similar to various other plant-based inhibitors such as tea extract and Asian arrowroot leaf extract [56,57]. Additionally, the anodic and cathodic branches’ linearity indicates that both reactions are controlled by a charge transfer process [58].

With respect to the corrosion current density, the result revealed that the values reduced appreciably upon the increase of EFB-H dosage. The corrosion current density is related to the electrochemical reaction rate and can be easily determined experimentally by extrapolating the Tafel plot [59]. Lower corrosion current density is preferred upon addition of inhibitor as it indicates a lower corrosion rate. Based on the result, the reduction of current density and corrosion rate reflects that the inhibitor molecules were adsorbed on the mild steel surface and retarded the electrochemical reactions [60]. Hemicellulose components such as xylose, arabinose and glucose are among the inhibitor molecules adsorbed. Other molecules in EFB-H might as well be adsorbed on the surface; however, due to the low concentration, their contribution to the inhibition is not superior. The inhibition efficiency resembles the inhibitor’s performance, which was calculated from the reduction of the current density. According to the result, it is evident that the inhibition efficiency increased with the increase of EFB-H dosage. This indicates that more inhibitor molecules are adsorbed at higher dosages, providing better surface coverage and better inhibition performance [60].

#### 3.2.2. Electrochemical Impedance Spectroscopy

**Figure 6:**
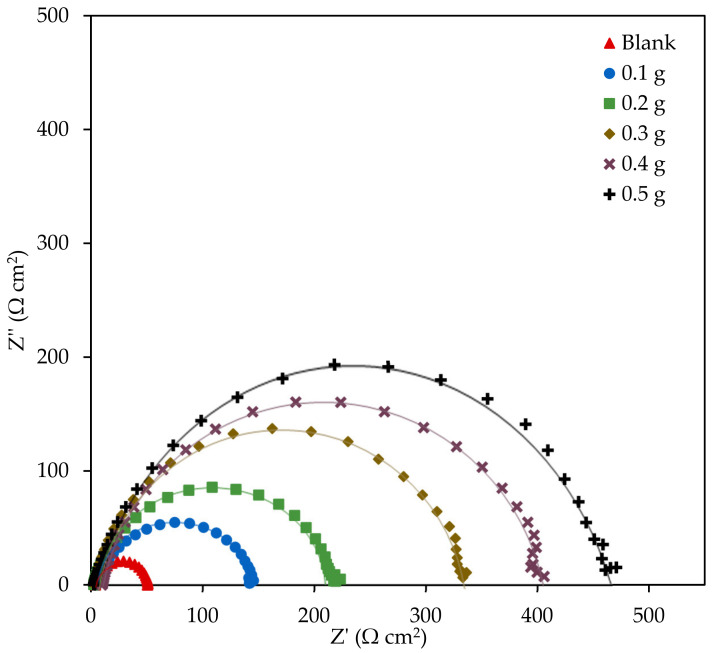
Nyquist plots of mild steel in 1 M HCl at different EFB-H dosages.

To procure a more detailed information on the corrosion inhibition mechanism of EFB-H on mild steel, EIS experiment was also performed. In the EIS method, impedance is used to relate theoretical circuit elements to the corrosion system [61]. The Nyquist plots of mild steel in the absence and presence of inhibitor are illustrated in Figure 6. Distinctly, the plots comprised of single capacitive loops, which suggests that the corrosion process is controlled by a charge-transfer process [62,63,64]. However, the capacitive loops curve slightly deviated from a perfect semicircle shape, especially at higher frequencies. In addition, the curves are also slightly depressed downwards instead of having perfectly round curvatures. These discrepancies are predominantly caused by the mild steel surface’s heterogeneity and roughness, which is a typical condition of the corrosion system [27]. With respect to the capacitive loop diameter, the size of the uninhibited 1 M HCl was the smallest, and larger loops were obtained as the EFB-H dosage was increased from 0.1 to 0.5 g. The diameter of the loop resembles the charge transfer resistance, *R_ct_*. The larger diameter of the capacitive loops at higher inhibitor dosage implies higher resistance to corrosion due to more molecules’ adsorption on the mild steel surface [27].

To further understand the electrochemical aspect of the corrosion inhibition, the results from EIS measurement were fitted to an equivalent circuit. Ideally, the most straightforward Nyquist plot with only one perfect semicircle can be simulated by Randles equivalent circuit, which assumes homogeneous and flawless metal surface that behaves as a pure capacitor. However, in reality, the metal surface is microscopically rough and heterogeneous even with extensive cleaning and polishing. Accordingly, a constant phase element (CPE) was employed instead of a pure capacitor to accurately resemble the data [65]. The electrochemical parameters such as the solution resistance, charge transfer resistance, factor of deviation due to metal roughness and double layer capacitance in addition to the inhibition efficiency are listed in Table 5.

In terms of the solution resistance, no obvious trend was observed in blank and inhibited solutions. Thus, it can be inferred that EFB-H do not alter the ionic conductivity of the solution. The charge transfer resistance resembles the metal-solution interface’s resistance that prevents the movement of ions from the metal into the aqueous solution. As can be seen in the table, the charge transfer resistance increased by 3 folds from 47.28 to 142.08 Ωcm^2^ upon the addition of OPEFB waste hydrolysate at the lowest dosage. With successive increases in the inhibitor dosage, a substantial increment of resistance was observed as anticipated. By utilizing the reduction of charge transfer resistance, the inhibition efficiency of EFB-H can be calculated. The finding is in agreement with the inhibition efficiencies obtained by weight loss and polarization techniques, which demonstrated that inhibition efficiency improved with increasing inhibitor dosages.

The deviation factor ranging from 0 to 1 indicates the deviation of the actual situation from the ideal system. A value closer to 0 implies that the CPE behaves as a resistor, while a value closer to 1 suggests that the CPE acts similar to a capacitor, as proposed in Randles equivalent circuit [8]. Based on the data, the average deviation factor value was 0.998, which suggests that the mild steel corrosion in the presence and absence of EFB-H deviated by only 2% from the ideal circuit. The deviation observed in the study is far lower than those reported in the literature [8,27,66,67]. Hence it is possible to deduce that the mild steel surface inhomogeneity used in the study was not severe.

The double layer capacitance is a capacitance value associated with the double layer formed within the metal/electrolyte interface. Physically, the double layer capacitance exists in a corrosion system due to the charge distribution of ions and polar molecules such as water in the interface. According to the result, the highest capacitance of 145.36 µF cm^−2^ was observed in the blank solution, and appreciable capacitance reduction was observed as EFB-H was added into the solution. The decrease in capacitance value is typically caused by the reduction of the dielectric constant (*ε*) and/or the thickening of the double layer. Organic compounds typically have lower dielectric constant values (*ε* ≈ 3–4) compared to water (*ε* ≈ 80), owing to their outstanding electrical insulating properties [68,69]. Since the molecules of EFB-H displace water molecules on the metal surface upon adsorption, the overall electric conductance drops, and thus restricting the electrical current flow through the double layer [62]. Furthermore, the adsorption of larger inhibitor molecules will also increase the double layer’s thickness [70,71]. On that basis, it can be concluded that both aspects; namely the reduction of the dielectric constant and the thickening of the double layer, synergistically lowered the double layer capacitance and the corrosion reaction of the mild steel. 

### 3.3. Optimization—Response Surface Methodology

An inhibitor’s inhibition efficiency is immensely dependent on the inhibitor dosage and immersion time [72]. Hence, RSM/CCD optimization was performed to determine an optimum value of dosage and immersion time for maximum efficacy of EFB-H. Table 6 demonstrates the preliminary study result. It appeared that 0.3 g of EFB-H yielded the maximum inhibition efficiency, and higher dosages than 0.3 g had no significant influence on the inhibition efficiency. The optimum point of dosage at 120 h of immersion time possibly lied between 0.3 and 0.4 g of dosage. With respect to the immersion time, the efficiency improved as the immersion time was increased from 24 to 120 h. However, the performance declined as the corrosion test was prolonged to 216 h. The possible optimum point at 0.3 g of dosage was at 120 h of immersion time. Based on the findings, it can be deduced that the optimum point lies between the preliminary variables ranges; 0.1 to 0.5 g of EFB-H dosage and 24 to 216 h of immersion time. Hence this range would be appropriate for RSM optimization purpose. 

After determining the appropriate range of variables, RSM/CCD was performed to achieve optimum parameters for maximum inhibition efficiency. A total of 13 runs were generated in the design matrix, as depicted in Table 7. The expression representing the influence of EFB-H dosage and immersion time towards the inhibition efficiency of mild steel in 1 M HCl at 25 °C is given as follows:

(7) $$IE\;\left(\%\right)=44.98+55.82\;A+0.50\;B\;-\;5.73{\;\times \;10}^{-3}\;AB\;-\;69.44{\;A}^{2}\;-\;2.08{\;\times \;10}^{-3}\;B^{2}$$

where *IE* is inhibition efficiency, *A* is EFB-H dosage and *B* is immersion time.

**Figure 7:**
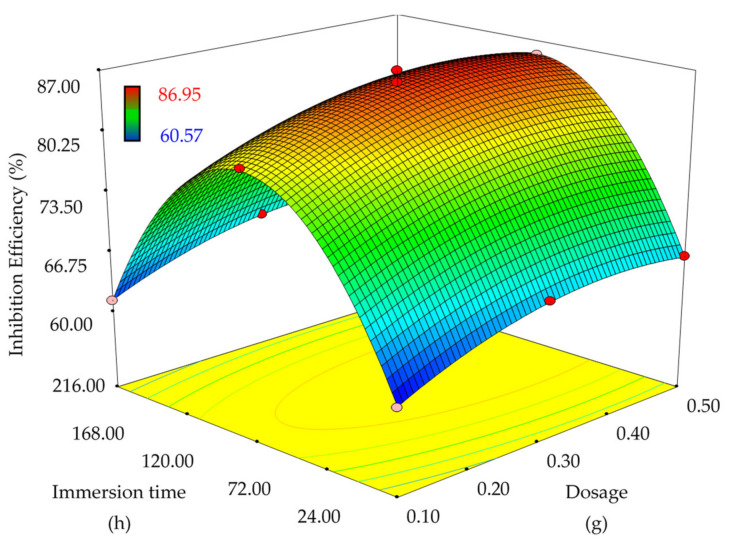
RSM surface plot; inhibition efficiencies of mild steel in 1 M HCl at different EFB-H dosages and immersion times at 25 °C.

Figure 7 depicts the response surface plot of EFB-H dosage and immersion time effects on the corrosion inhibition efficiency. It is evident that the inhibitor performance increased with increasing dosage from 0.1 to 0.3 g. Further increment of dosage to 0.5 g had no substantial effect on the performance. With respect to the immersion time, extending the immersion time from 24 to 120 h had increased the performance. This finding broadly supports the studies of other inhibitors such as galactomannan (1 to 48 h), benzimidazole derivative (24 to 72 h), Schiff base molecule (2 to 96 h) and carbon dots (6 to 120 h) [73,74,75,76]. Contrarily, the effectiveness of EFB-H reduced significantly beyond 120 h of immersion time. The decline in inhibition efficiency is rarely reported in the literature, probably due to the short time range (less than 120 h) used in the previous studies. Some studies had shown that the hydrolysis degradation of inhibitor molecules might occur during corrosion testing in acid solution [77]. Even though exhibiting a steep decline at prolonged immersion time, the EFB-H behaved excellently at 120 h, which is comparatively longer than other inhibitors.

The final part addresses optimizing the parameters for maximum inhibition efficiency of EFB-H using the generated model. For this purpose, the response (inhibition efficiency) was set to “maximum” to achieve the highest possible value. Based on the specified condition, the maximum efficiency was predicted to be 85.95% at an inhibitor dosage of 0.33 g and an immersion time of 120 h. A validation experiment at the optimum point revealed that the inhibition efficiency was 87.11 ± 1.91%, which proved that the model is accurate and reliable for prediction. Such a conclusion was made on the basis of a small error between the actual and the predicted value, which was only 1.35%.

### 3.4. Corrosion Inhibition Study of Oil Palm Empty Fruit Bunch Tablet Inhibitor

#### 3.4.1. Mixture Design Formulation

As discussed earlier, the effectiveness of EFB-H reduced after 120 h of immersion time, which is possibly caused by the desorption of inhibitor molecules and the hydrolysis degradation of inhibitor molecules in acid solution. In an attempt to prolong the effective duration of the inhibitor, a well-established concept in the pharmaceutical field, a sustained release tablet is applied herein as a proof of concept for enhancements in corrosion inhibition application. In addition, corrosion inhibitor tablets’ application is industrially advantageous in the sense of ease of handling, eliminating the need for regular dosing and ensuring a safer working environment [14]. In this study, EFB-H tablets incorporated with a polymer matrix with a diameter of 13 mm and a weight of 0.5 g were fabricated. The tablets were composed of an active ingredient: EFB-H in powder form, as well as individual and mixtures of two polymer binders, GA and HPMC. The formulations and the respective corrosion inhibition efficiency responses are tabulated in Table 8. The composition of EFB-H and the immersion time was controlled at the optimum condition derived from RSM optimization, which are at 66% (0.33 g per 0.5 g tablet) and 120 h.

The expression representing the effect of GA and HPMC composition towards the inhibition efficiency EFB-H tablet for mild steel in 1 M HCl at 25 °C is given as follows:

(8) $$IE\;\left(\%\right)=2.39\;C+2.47\;D\;-\;0.02\;CD$$

where *IE* is the inhibition efficiency, *C* is the composition of GA and *D* is the composition of HPMC, respectively. When dealing with formulations of a two-component mixture or more, in this case, GA and HPMC, it is worth to know if the components are working synergistically (better together) or antagonistically (better alone). Based on the last coefficient of Equation (8) (−0.02), it seemed that the polymers exhibited antagonistic effect [78]. 

**Figure 8:**
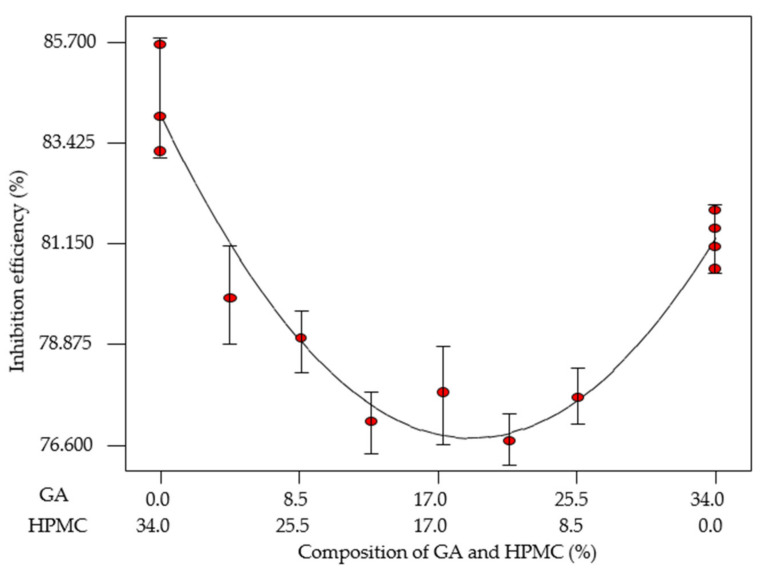
Two-component mixture plot; inhibition efficiencies of mild steel in 1 M HCl at different EFB-H tablet formulations at 25 °C.

The response trace plot of various GA and HPMC combinations is depicted in Figure 8 to represent the correlation. As evident from the plot, the best inhibition efficiency was obtained at the extreme ends of the formulation, whereby only a single polymer is incorporated in the tablet. With both polymers’ combination, the tablets showed gradual inhibition efficiency reduction. This reduction is an indication that GA and HPMC do not work very well together and is said to have an antagonistic effect on the performance [78]. Accordingly, only the best two formulations were selected for further investigations.

#### 3.4.2. Tensile Strength, Disintegration Time and Dissolution Profile

In the previous section, the effect of different tablet formulations on the corrosion inhibition efficiency was investigated, and two best tablet formulations were determined (T2 and T3). Those two tablets, as well as a control tablet (T1) were further investigated in terms of their tensile strength, disintegration time and dissolution profile. The tensile strength is an essential mechanical property to determine the tablets’ integrity. It is greatly affected by the excipients used in the formulation and their particle size distribution [79]. The tensile strengths of tablet T1, T2 and T3 are compiled in Table 9. As evident from the data, the highest strength was exhibited by T3 (0.243 MPa), followed by T1 (0.130 MPa) and T2 (0.099 MPa). T2 that was incorporated with GA exhibited inferior mechanical strength. The low strength is probably due to GA’s brittleness, which caused breakage within the compressed powder, hence damaging the tablet’s overall structure [80]. On the other hand, the addition of HPMC in the formulation improved the tensile strength of T3 by more than twofold compared to the control. This result is consistent with those of Siow et al. [81] who found out that incorporating HPMC showed positive contribution to mannitol tablets’ strength. 

In terms of the tablets’ disintegration time in 1 M HCl media, T3 showed a longer disintegrating period than T1 and T2. This finding indicates that HPMC better facilitates the slower release of EFB-H to the corrosive solution. HPMC is excellent in slowing down the disintegration due to its high hydrophilicity, enabling the polymer to swell and become gelatinised upon contact with water [82]. The formation of the polymer gel retards the diffusion of active ingredients and the tablet’s erosion. From the corrosion inhibition perspective, the slow release of inhibitor compounds from a matrix will lead to sustained service life of an inhibitor [83].

**Figure 9:**
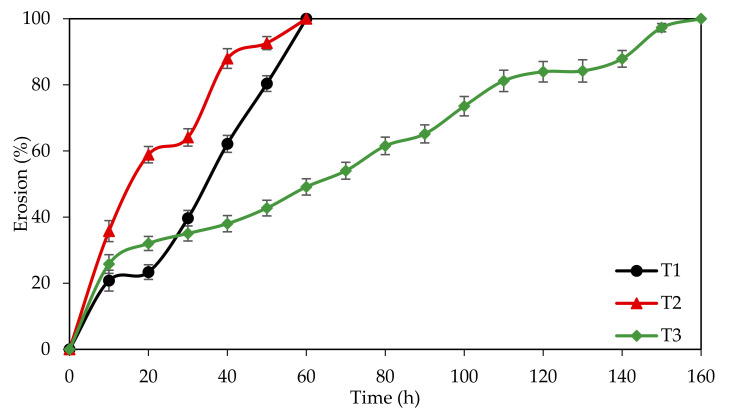
Dissolution profiles of T1, T2 and T3 in 1 M HCl at 25 °C and 100 rpm of agitation speed.

The dissolution profile is also an essential property of a corrosion inhibitor tablet as it is crucial to have a sufficient and continuous supply of inhibitor over time to avoid localised corrosion. The dissolution profiles of T1, T2 and T3 are illustrated in Figure 9. Evidently, the plot revealed that each tablet’s dissolution behaviour was undoubtedly distinct from each other. A linear dissolution trend is observed for the control tablet starting from the initial time up to 10 min and from 20 min to 60 min in the media (T1). This constant supply of inhibitor explains the excellent corrosion inhibition of T1. For the case of T2, the dissolution rate was not uniform, which starts relatively faster than T1 and T3, and fluctuates starting from 20 to 60 min of immersion. This disintegration manner is quite risky for corrosion inhibition application. The first reason is that with the rapid release of the inhibitor, the molecules degrade faster, and hence it will not be efficient in the long run [77]. Secondly, with delayed inhibitor release, the concentration might fall below the minimum at a certain time. For the case of EFB-H that is a mixed type inhibitor, lower concentration might induce localised or pitting corrosion in some area where the surface is not fully covered by the inhibitor compounds [84]. T3 showed the best dissolution action among all the tablets, which was gradual until it completely dissolves. Even though showing some slight delay in disintegration at the 120th minute, the overall behaviour is still better than T1 and T2. Similar dissolution behaviour of other HPMC incorporated tablets such as mineral salt tablet and mannitol tablet were reported in the literature [81,85].

In general, based on the tensile strength, disintegration time and dissolution profile, T3 portrayed the best performance, followed by T1 and T2. The addition of polymer in the EFB-H tablet had modified the tablet properties substantially. Despite the promising results, the tensile strength of T3 is still meagre (0.243 MPa) compared to other HPMC bound tablets reported elsewhere (0.16 to 1.75 MPa) [81,86]. Hence, improvements should be made to enhance the tablet strength further. Moreover, the dissolution test conducted in the study is based on a standard procedure meant for pharmaceutical application. Even though beneficial for this preliminary study, a more specific method for corrosion inhibition application should be developed for more accurate result. Additionally, the likelihood of added polymers participating in the corrosion inhibition mechanism cannot be ruled out as numerous studies have demonstrated that polymers, such as GA, are excellent corrosion inhibitors. Since there is hardly any reported application of compressed tablets in the field of corrosion inhibition except for one patented technology [14], further work is required to address this matter. In addition, there are various other properties that should be considered, but only the crucial parameters relevant for corrosion inhibition are investigated at this stage.

#### 3.4.3. Extended Weight Loss Study

**Figure 10:**
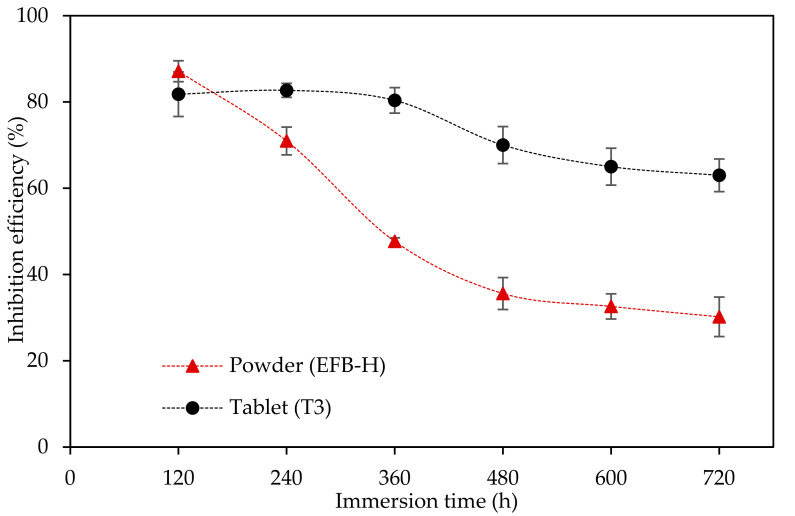
Inhibition efficiencies of mild steel in 1 M HCl at different forms of EFB-Hs at different immersion times at 25 °C.

To prove the EFB-H tablet’s effectiveness at an extended time, a prolonged weight loss study (120 to 720 h) for both powder and tablet forms of EFB-H was performed. T3 formulation was selected for this examination due to its excellent properties, as discussed in the previous section. The trends of the inhibition efficiency of T3 and EFB-H powder at extended immersion times are illustrated in Figure 10.

As expected, EFB-H powder showed a reduction in inhibition efficiency after 120 h of immersion. Contrarily, the tablet showed nearly persistent performances over 360 h despite having slightly lower efficacies relative to the powder at 120 h of immersion time. The longer inhibitive time of the tablet is explicable to the sustained release of EFB-H active compounds. The gradual release prevents desorption and degradation of the inhibitor molecules, which are the leading causes of efficacy reduction at longer immersion time [10,77,87]. Beyond 360 h, the tablet exhibited some inhibition efficiency reduction, but it was not as extensive as portrayed by the powder. The depletion of inhibitor compounds possibly causes a decrease in performance. A commercial tablet for an industrial water treatment system that includes polymers, biocide, surfactant, scale inhibitor and corrosion inhibitor in the formulation with a bigger tablet diameter, approximately 70 mm, could hold up to six months [14]. Therefore, increasing the dose of EFB-H and the size of the tablet may extend the shelf life of the EFB-H tablet for longer time.

On the basis of this finding, it can be deduced that the sustained release tablet has a longer-lasting inhibition effect than the powder. Therefore, the implementation of a well-acknowledged pharmaceutical technology in corrosion engineering is proven to be successful. While preliminary, this finding leads to a number of possibilities and offers a revolutionary step in the industrialisation of nature-based corrosion inhibitors, which is a huge and unbridged gap between research and commercialisation [88].

## 4. Conclusions

In general, this project aimed to develop a controlled release EFB-H inhibitor tablet to inhibit mild steel acid corrosion. This study contemplated the conventional fundamental study of a corrosion inhibitor as well as its engineering application. Based on the findings, it can be summarized that the presence of heteroatoms and reactive functional groups in EFB-H has been validated through FTIR analysis, and 89.51% of hemicellulose was quantified via compositional analysis. In addition, the PDP measurement has shown that EFB-H inhibited mild steel corrosion by a mixed mechanism of anodic and cathodic reactions retardation. A reduction of current density and corrosion rate was observed as the dosage was increased, reflecting that more inhibitor molecules are adsorbed at higher dosages. From EIS analysis, it has been revealed that EFB-H increased the charge transfer resistance from metal to the solution and reduced the double layer capacitance within the metal/electrolyte interface. Furthermore, it was discovered that the corrosion inhibition system could be integrated into a modified Randles circuit with a CPE. RSM/CCD optimization has unveiled that the optimum point for a maximum inhibition efficiency (87.11%) was at 0.33 g of EFB-H dosage and 120 h of immersion time. Regarding tablet development, T3 with EFB-H:GA:HPMC ratio of 66:0:34 portrayed the best tensile strength (0.243 MPa), disintegration time (152 min), dissolution behavior and adequate corrosion inhibition performance (84.30%). In comparison with EFB-H in powder form, the EFB-H tablet exhibited a longer-lasting inhibition effect, which was 360 h compared to 120 h for powder. Overall, these results suggest that the EFB-H tablet has been successfully developed, and its enhanced effective inhibition time has been experimentally proven. The innovative approach of developing a controlled release inhibitor tablet may have unraveled the emerging issues when dealing with natural inhibitors. This pioneering discovery provides a colossal foundation to utilize nature-based inhibitors for commercial applications. The precise inhibition mechanism of EFB-H remains to be elucidated. Hence, a study that assesses the inhibition mechanism through adsorption isotherm study, comprehensive chemical characterization, quantum chemical calculation and molecular dynamic simulation will need to be carried out in the future.

## Figures and Tables

**Table 1:** Chemical composition of mild steel.

Element	Chemical Composition (wt.%)
Iron	98.874
Carbon	0.140
Silicon	0.170
Manganese	0.550
Copper	0.230
Sulfur	0.027
Phosphorus	0.009

**Table 2:** Tablet formulation of T1, T2 and T3.

Tablet	Tablet Formulation (% *w*/*w*)		
	EFB-H	GA	HPMC
T1	100.0	0.0	0.0
T2	66.0	34.0	0.0
T3	66.0	0.0	34.0

**Table 3:** Chemical compositions of raw OPEFB and EFB-H.

Components	Composition (% *w*/*w*)	
	Raw OPEFB	EFB-H
Holocellulose	83.56	90.34
Cellulose	29.85	0.85
Hemicellulose	53.71	89.51
Lignin	15.64	9.28
Acid soluble	8.78	5.39
Acid insoluble	6.86	3.89
Extractives	0.81	0.38

**Table 4:** Potentiodynamic polarization parameters of mild steel in 1 M HCl at different EFB-H concentrations.

EFB-H Concentration (g 300 mL^−1^)	*E_corr_* (mV)	*β_a_*	*−β_c_*	*I_corr_* (µA cm^−2^)	*C_R_* (mm y^−1^)	*IE_PDP_* (%)
		(mVdecade^−1^)				
Blank	−463.51	122.14	135.25	598.65	8.79	-
0.1	−420.14	129.45	128.31	163.15	2.40	72.75
0.2	−418.84	136.97	145.08	128.28	1.88	78.57
0.3	−431.57	109.04	139.38	78.04	1.15	86.96
0.4	−421.95	109.59	133.09	58.52	0.86	90.22
0.5	−414.49	111.89	140.28	60.31	0.89	89.92

Note: *E_corr_* is corrosion potential; *β_a_* is anodic Tafel slope; *β_c_* is cathodic Tafel slope; *I_corr_* is corrosion current density; *C_R_* is corrosion rate; *IE_PDP_* is inhibition efficiency from potentiodynamic polarization method.

**Table 5:** Electrochemical impedance parameters of mild steel in 1 M HCl at different EFB-H concentrations.

EFB-H Concentration (g 300 mL^−1^)	*R_s_* (Ω cm^2^)	*R_ct_* (Ω cm^2^)	*IE_EIS_* (%)	*n*	*C_dl_* (µF cm^−2^)
Blank	3.78	47.28	-	0.9987	145.36
0.1	4.06	142.08	66.72	0.9971	93.20
0.2	3.18	216.14	78.12	0.9975	83.56
0.3	1.68	336.13	85.93	0.9978	72.81
0.4	1.20	397.11	88.09	0.9978	69.07
0.5	4.09	462.77	89.78	0.9979	72.86

Note: *R_s_* is solution resistance; *R_ct_* is charge transfer resistance; *IE_EIS_* is inhibition efficiency from EIS method; *n* is deviation factor; *C_dl_* is double layer capacitance.

**Table 6:** Inhibition efficiencies of mild steel in 1 M HCl at different EFB-H dosages and immersion times at 25 °C using OFAT method.

Independent Parameter		*IE_WL_* (%)
EFB-H Dosage (g)	Immersion Time (h)	
0.1		80.37 ± 0.72
0.3	120	85.66 ± 1.28
0.5		85.37 ± 1.44
	24	66.35 ± 0.69
0.3	120	85.66 ± 1.28
	216	66.69 ± 1.43

Note: *IE_WL_* is inhibition efficiency from weight loss method. Values are mean ± standard deviation.

**Table 7:** CCD matrix of two variables (EFB-H dosage and immersion time) and one response (inhibition efficiency).

Standard Order	Run Order	EFB-H Dosage (g)	Immersion Time (h)	*IE_WL_* (%)
1	10	0.1	120.0	80.37
2	4	0.5	120.0	85.00
3	8	0.1	360.0	59.02
4	6	0.5	360.0	77.61
5	2	0.1	240.0	57.25
6	11	0.5	240.0	66.55
7	9	0.3	120.0	85.66
8	1	0.3	360.0	77.41
9	7	0.3	240.0	65.23
10	13	0.3	240.0	67.89
11	5	0.3	240.0	67.84
12	3	0.3	240.0	66.49
13	12	0.3	240.0	64.57

Note: *IE_WL_* is inhibition efficiency from weight loss method.

**Table 8:** D-optimal mixture design matrix for two mixtures (GA and HPMC) and one response (inhibition efficiency).

Standard Order	Run Order	EFB-H (%)	GA (%)	HPMC (%)	*IE_WL_* (%)
1	1	66.00	34.00	0.00	81.90
2	3	66.00	0.00	34.00	85.64
3	7	66.00	17.34	16.66	80.79
4	13	66.00	8.62	25.38	79.01
5	11	66.00	25.62	8.38	77.67
6	10	66.00	12.94	21.06	77.12
7	6	66.00	4.29	29.71	79.92
8	4	66.00	21.42	12.58	76.69
9	5	66.00	34.00	0.00	81.07
10	2	66.00	0.00	34.00	84.02
11	9	66.00	34.00	0.00	80.57
12	12	66.00	0.00	34.00	83.23
13	8	66.00	34.00	0.00	81.49

Note: *IE_WL_* is inhibition efficiency from weight loss method.

**Table 9:** Formulations, inhibition efficiencies and tablet properties of selected tablets.

Tablet	Formulation (% *w*/*w*)			*IE_WL_* (%)	Tensile Strength (MPa)	Disintegration Time (min)
	EFB-H	GA	HPMC			
T1	100.0	0.0	0.0	87.12	0.130 ± 0.027	63.00 ± 1.00
T2	66.0	34.0	0.0	81.28	0.099 ± 0.017	53.00 ± 3.00
T3	66.0	0.0	34.0	84.30	0.243 ± 0.006	152.00 ± 2.00

Note: *IE_WL_* is inhibition efficiency from weight loss method; Values are mean ± standard deviation.

## Data Availability

Data available on request from the authors.
